# Discrimination and Elder Abuse Toward Elderly LGBT Individuals:A Systematic Review

**DOI:** 10.1192/j.eurpsy.2025.1767

**Published:** 2025-08-26

**Authors:** P. Yılmaz, C. H. Ayhan, H. Akbulut, Ö. Sukut

**Affiliations:** 1 Sağlık Bilimleri Fakültesi, Van Yüzüncü Yıl Üniversitesi, Van; 2 Sağlık Bilimleri Fakültesi, İstanbul Üniversitesi, istanbul, Türkiye

## Abstract

**Introduction:**

Despite increasing social acceptance over the past two decades, lesbian, gay, bisexual, and transgender (LGBT) individuals continue to face unequal treatment in society (Institute of Medicine, 2011; Caceres et al., 2020). Elderly LGBT adults include the population of sexual and gender minorities over the age of 50 (Choi et al., 2016).They continue their lives under the shadow of negative societal stereotypes and assumptions about their physical and mental health, sexuality, sexual lives, and ability to contribute to society, as well as their family and societal values. When individuals find themselves at the intersection of being elderly and LGBT, they encounter a combined situation that leads to an increased risk of discrimination, social exclusion, and violence.Over time, this can lead to high levels of depression, anxiety, loneliness, and other mental health issues. When they are not accepted by social circles, it often results in social isolation and loneliness. (Geneva, 2023)

**Objectives:**

The aim of this study is to examine the challenges faced by elderly LGBT individuals by addressing issues of discrimination and elder abuse. By evaluating the extent to which current services meet the needs of these individuals, it aims to propose solutions from the perspective of social equality and human rights.

**Methods:**

The study was conducted between December 2024 and February 2025 using the keywords “elderly,” “elderly LGBT individuals,” and “LGBT discrimination” “elder abuse” in databases (PubMed, Scopus, Springer, etc.). These databases have been preferred because they contain a significant amount of evidence-based literature in the field of psychology. Studies conducted between 2000 and 2024, with full texts accessible and written in Turkish and English, have been included in the study

**Results:**

As of November 2024, 28 national and international research articles related to the subject have been reached. The literature review is ongoing. When the literature review is completed, all the study results will be presented together.

**Conclusions:**

The human rights violations, discrimination, social exclusion, and risk of violence faced by elderly LGBT individuals are not only individual but also societal issues.Developing policies that are sensitive to the needs of elderly LGBT individuals in social services, healthcare services, and the legal system is an important step toward improving their physical, mental, and social health.Awareness-raising activities need to be expanded to reduce stigma and discrimination against elderly LGBT individuals in society. Additionally, establishing LGBT-friendly elderly care centers and strengthening social support networks are crucial to preventing social isolation.In conclusion, achieving a more inclusive, equal, and fair society is only possible by respecting the rights of every individual.

**Disclosure of Interest:**

None Declared

